# Spatio-temporal determinants of mental health and well-being: advances in geographically-explicit ecological momentary assessment (GEMA)

**DOI:** 10.1007/s00127-016-1277-5

**Published:** 2016-08-24

**Authors:** Thomas R. Kirchner, Saul Shiffman

**Affiliations:** 1 College of Global Public Health, New York University, 41 E. 11th St., 7th Floor, New York, NY 10003 USA; 2 Center for Urban Science and Progress, New York University, New York, NY USA; 3 Department of Population Health, New York University Medical Center, New York, NY USA; 4 Department of Psychology, University of Pittsburgh, Pittsburgh, PA USA; 5 Department of Psychiatry, University of Pittsburgh Medical Center, Pittsburgh, PA USA; 6 Department of Pharmaceutical Sciences, University of Pittsburgh School of Pharmacy, Pittsburgh, PA USA; 7 Clinical and Translational Science Institute, University of Pittsburgh, Pittsburgh, PA USA

**Keywords:** Ecological momentary assessment (EMA), Geographic momentary assessment (GMA), Geographically explicit ecological momentary assessment (GEMA), Geographic information systems/science (GIS), Spatio-temporal determinants of health, mHealth

## Abstract

**Purpose:**

Overview of geographically explicit momentary assessment research, applied to the study of mental health and well-being, which allows for cross-validation, extension, and enrichment of research on place and health.

**Methods:**

Building on the historical foundations of both ecological momentary assessment and geographic momentary assessment research, this review explores their emerging synergy into a more generalized and powerful research framework.

**Results:**

Geographically explicit momentary assessment methods are rapidly advancing across a number of complimentary literatures that intersect but have not yet converged. Key contributions from these areas reveal tremendous potential for transdisciplinary and translational science.

**Conclusions:**

Mobile communication devices are revolutionizing research on mental health and well-being by physically linking momentary experience sampling to objective measures of socio-ecological context in time and place. Methodological standards are not well-established and will be required for transdisciplinary collaboration and scientific inference moving forward.

## Introduction

The stuff of the mind is the stuff of the world, and so the investigation of the rich structure of the world provides a clearly observable and empirically tractable—if not royal—road into the hidden countries of the mind.

Tooby & Cosmides, 1992

The Psychological Foundations of Culture

Lifespan disparities in excess of 10 years between residents of neighborhoods characterized the lowest versus the highest levels of socio-economic status (SES) have persisted in the United States for decades [1, 2]. Findings from three large longitudinal studies [2–5], together spanning the period from 1979 to 2014, indicate that lifespan disparities are largely due to neighborhood residents’ moment-to-moment mental health and well-being, insofar as these factors drive behavioral decisions—whether to exercise or smoke cigarettes, what to eat and drink, and whether to continue pursuing an education. These studies also converge to suggest that neighborhood characteristics promote and protect against these behavioral health determinants—especially within low-SES areas. Adjusting for a range of demographic factors, Chetty et al. [1] found that low-income residents of otherwise affluent and highly educated cities such as New York and San Francisco were *protected* from the loss of as many as 5-years of life relative to low-income residents of less-affluent cities such as Detroit, Michigan or Gary, Indiana. The mechanisms behind this finding are undoubtedly complex, exposing how little we know about the way neighborhoods shape subjective experience and ultimately determine the psychological and physical health of their residents.

This paper presents an overview of the way ecological momentary assessment (EMA [6–9]) methodologies have been used to study the interplay between individuals’ experience and their evolving environmental surroundings, with specific emphasis on the measurement of mental health and well-being. With traditional roots in real-time experience sampling [10–12], EMA primarily involves administration of self-reported survey items in real-life settings (i.e., collected as part of subjects’ ongoing activities of daily living). More recently, technological advances have led to the development of geographic momentary assessment (GMA) methods, which are founded on the notion that individuals can be characterized by a spatio-temporal probability distribution—time spent amongst a set of places, each with their own geographic boundaries and temporal characteristics (e.g., duration and cyclic regularity of visits) [13–16]. Combined within a common, geographically explicit ecological momentary assessment (GEMA) framework,^1^ EMA and GMA dovetail with a number of socio-ecologic theoretical models [18–23]—together providing an explicitly geographic measurement framework that can be leveraged to study the way people and places reciprocally determine each other over time [24]. This GEMA framework is more than the raw Cartesian coordinates that distinguish it from traditional EMA, and is often most useful for the study of “places” imbued with meaning by subjects—representing each subject’s personal eco-system, itself populated by an array of spatially weighted geographic correlates with known socio-contextual significance.

## Neighborhoods to neurons: ecological momentary assessment of mental health

The rise of EMA methodology in the behavioral health sciences is largely due to the fact that it documents subjective experiences, including symptoms of mental distress, in natural environments. The primal brain structures designed to protect us from threats via affective signals like fear, disgust, and anger evolved within ancestral environments [21, 25, 26]. The adapted human mind is preprogrammed to identify fearful stimuli, and given that emotional reactivity becomes conditioned under chronic exposure to environmental conditions, cultural anthropologists and environmental psychologists have written extensively on the idea that study of structural and cultural environments can improve our understanding of mental health [27–30]. This includes the evolution of addictive and maladaptive eating behaviors, which are exacerbated by modern “toxic” environmental conditions, often intentionally designed to cue deeply ingrained desires for consumption [31, 32].

Standard diagnostic practice within psychiatry and clinical psychology is based on structured psychometric interviews that assess the frequency and severity of affective and behavioral symptoms over a specified period of time—generally the past 2-weeks for acute symptoms. This approach requires respondents—sometimes in acute distress—to describe complex patterns of symptom expression. A limitation of this is that retrospective recall often diverges from reality, as evidenced by EMA research on symptom fluctuation [33, 34], and mobile interventions [35–38]. Clinical researchers have utilized EMA to study a range of psychopathological symptom profiles. Most work has focused on symptoms related to additive disorders [8] or depression [39, 40], but studies have also assessed symptoms in patients suffering from anxiety [41], schizophrenia and psychosis [42–44], chronic pain [45], attention deficits [46], eating disorders [47], and personality disorders [48].

An ongoing challenge is that while symptom variation across socio-ecological circumstances is at least as important as their basic frequency and duration, self-reported EMA surveys are not well-suited for systematic documentation of symptom fluctuation over both time and place. EMA protocols often include survey items that assess certain socio-ecologic circumstances, but the full range of structural and cultural factors that could be involved extend well beyond what an individual could be expected to perceive, much less report with fidelity (e.g., ambient light and air quality; neighborhood disorder; local social norms) [49]. Even if complete information were available to respondents, the affective neuroscience literature now strongly supports Zajonc’s (1980 [50]) dictum that “preferences need no inferences”, suggesting that humans generally lack conscious, introspective awareness of the cognitive underpinnings of their feelings [51–53].

Geographically explicit EMA (GEMA) integrates EMA with GMA methods and thus geographic information system (GIS) science, allowing for cross-validation and enrichment of research on place, well-being, and health. This is accomplished with mobile geographic location technologies that physically link participants to their current position in two-dimensional space. This geographic link operationalizes the “ecological” aspect of EMA, such that geographic locations can be quantified precisely and then attached to corresponding sources of socio-ecologic data. Setting the stage for this methodological integration, health-related GMA research has advanced rapidly over the past 10-years, including considerable work that focuses on momentary measurements of individuals’ geographic location.

## Geographic momentary assessment (GMA)

### Mere mobility

The physical touch-point between individuals and their surroundings and thus the foundational nexus of all person-place dynamics is their geographic location—shifting over time between periods of stability and periods of movement. The basic science of human mobility extends back through decades of work on international migration and macro-economic trends [54, 55], and has recently been reignited by new geographic location technologies and resultant sources of data. Even with as little as the timing and location of cell-tower transmissions, for example, computational social scientists have shown that passive observation of call-data records from cellular telephone providers can be used to predict individual movement patterns and even identify social networks [56–59]. This important work is establishing precise metrics for the study human mobility, although it suffers in some ways from an aggregated, macro perspective some have used to question its societal impact [60, 61].

The use of geographically explicit human mobility data for the study of health was pioneered around the turn of the century by environmental exposure scientists interested in individual-level patterns of exposure to air and water pollution [62–69]. While exposure science work of this kind has focused almost exclusively on biological exposures to environmental pathogens—sometimes referred to as the “exposome”—there is growing recognition that linking purely chemical conceptions of the exposome to the broader ecosphere or “eco-exposome” can foster transdisciplinary perspectives and possibly novel insights regarding health and place [70–72]. For example, behavioral scientists interested in effects of the built environment on physical activity levels have also contributed a great deal to research on geographically explicit measures of mobility and place [73–81], and extensions of this work are beginning to link individual mobility data to a range of geographic features correlated with diet and drug-use [82–86].

While their eventual convergence seems inevitable, computationally oriented disciplines founded on big-data analytics are too often disconnected from disciplines considered the standard-bearers of research on place and health. A field that some refer to as spatio-temporal epidemiology has grown from roots in sociology, geography, public health, and statistics, [87, 88] while other well-established fields such as psychiatry, clinical psychology, and environmental exposure science continue to emphasize connections between socio-ecological circumstances and mechanisms operating below-the-skin [38, 70]. Division between disciplines notwithstanding, it is encouraging that all share strong conceptual and methodologic interest in the way that individuals’ geographic location meanders over time, providing the foundation for a common ecologic framework for the design and implementation of health research.

### Geographic correlates

Long before interest grew in the way street networks affect physical activity patterns, urban planners and sociologists had recognized the symbiotic relationship between pedestrian traffic and municipal development [89, 90]. For instance, Jane Jacobs described the way “short blocks” within cities determine the flow of residents and thus catalyze opportunities for retail businesses to emerge and grow [90]. Street network structures and the associated access that pedestrians have to risk and protective factors in their local area—often referred to as an area’s walkability—provides an example of the way geographically distributed determinants of health can be linked to the well-being and health of local residents [91, 92]. Even in the absence of data on individual travel routes, indices of walkability can be a useful approximation of potential opportunities for access, and can thus provide a means to quantify differential access to risk and protective factors within and between neighborhoods.

A useful example concerns walking routes to and from school, which has been identified as an important Industry vector for the sale of retail products. In New York City, which consistently ranks among the most walkable US cities, as many as 61 % of six graders walk or bike to school [92, 93]. As adolescent decision-making autonomy grows in combination with a developing system of attitudes, beliefs, and emotional self-regulatory control, adolescents’ point-of-sale purchase decision-making is heavily influenced by retail access and marketing [94–96]. For example, point-of-sale food options vary across neighborhoods [97, 98], and the location of neighborhood food sources has been associated with adolescents’ food intake [80, 99–101] and overweight status [102–107].

Most studies of this kind correlate cross-sectional survey data with neighborhood data from within an arbitrary geometric area around schools [108, 109], or within walkable activity-spaces estimated with spatial network analytics [103, 107, 110, 111]. Similar approaches have been used to examine associations between product consumption and the corresponding geographic density of retail tobacco [112–121] and alcohol outlets [122–124]. A key assumption here is that the spatial concentration of health-risk and protective factors sufficiently approximates and is thus representative of individual-level exposure levels. An alternative is that observed associations between product landscape densities and use patterns are not driven by individual-level point-of-sale interactions per se, but by unobserved, shared determinants of both product availability and use among those who reside in different neighborhoods [125].

Studies contrasting the uncertainty of GIS-based exposure estimates have found that approximated activity space methods do not correspond well with real-time exposure data [69, 80, 81, 126–129]. For example, our group has developed methods for direct comparison of retail product exposure estimates based on residential approximation versus continuous observation of geospatial contact [130, 131]. Results of one analysis indicate that as much as 55.2 % of the variance in real-time exposures was *not* explained by residential density levels, suggesting that purely residence-based analyses misclassify large amounts of exposure data. Thus, while latent conceptualizations of neighborhood walkability and risk factor access can enrich our understanding of health and place, caution must be taken when drawing inference about underlying mechanisms in the absence of data on actual person-place dynamics.

## Geographically explicit ecological momentary assessment (GEMA)

Whether and how exposures to retail products and other neighborhood risk and protective factors impact residents’ behavior remains poorly understood [101, 108]. Access to products may affect behavioral decision-making in a way that is incidental—operating by cueing spontaneous purchase decisions—or as a matter of convenience, influencing deliberate decisions to selectively seek out one product or another. One way to investigate this would be to examine actual mobility routes in conjunction with EMA purchase reports, in an attempt to identify cases wherein subjects deviate from their established routes when seeking products. Note that this leverages within-subject data to ascertain the geospatial context of product purchases and draw inferences about geographic correlates, an approach not possible with cross-sectional or macro-aggregated data. Alternatively, a mixed-methods approach that has subjects annotate a map of the places they visit could be used, alone or supplemented by EMA reports regarding intentional versus spontaneous purchases [132, 133].

Quantifying the interactive influence of geographically dispersed neighborhood factors on health is not possible with layered density approaches alone. Neighborhood walkability may increase activity and reduce overweight, while it might simultaneously expose kids to air pollution and retail products in a way that varies from neighborhood to neighborhood [65, 134]. Interactions of this kind happen at the individual level, and it is at that nexus point that multilevel influences can be contrasted more directly. A recent, policy-oriented example comes from Pearson et al., working in Washington, DC, who incorporated geographically explicit information on local smoke-free policies and socially imposed smoking restrictions alongside continuous GMA of participant locations and EMA reports of cigarette and electronic cigarette use [132].

A handful of research groups have implemented full-scale GEMA protocols for the study of addictive drug-use behaviors [82, 83, 132, 135–138]. Among the most consistent findings from this body of work is the central role played by motivational drive states like drug-use craving [82, 135, 136, 138, 139]. This supports the notion that neighborhood product density plays a direct role, triggering craving and thus purchase-decisions, rather than simply enabling purchases that were planned ahead of time. Considered within a broader socio-ecologic framework, a cued-craving model of neighborhood influence is likely an over-simplification, as the presence of craving implies an established and often firmly engrained pattern of use. It has been observed that predictors of behavioral maintenance and cessation are often different than predictors of initiation [140, 141], spawning new questions about the impact of neighborhood factors on drug-use initiation over the course of early childhood and adolescence.

Extending beyond the retail product landscape, GEMA can be used to construct a geo-social tapestry that includes local policies, park conditions, crime, and other systems level economic factors that cannot be captured by EMA alone. In a series of GEMA studies, Preston et al. supplemented standard measures of drug craving and use with information about neighborhood conditions and physiological correlates like stress [17, 142]. Findings extend other EMA work on precipitants of drug use, incorporating the ways that stressful neighborhood settings underlie and can moderate their effects [143, 144]. Regarding etiology, available evidence suggests that the formation of maladaptive, cued associations with a specific substance or product depends on self-regulatory processes [145–147], in the sense that individuals facing stressful environmental conditions are more likely to utilize coping resources that are readily available within the same ecologic system that exacerbated their distress in the first place.

Mobile communication devices will continue to revolutionize research on mental health and well-being by linking momentary experiences to objective measures that can be collected unobtrusively [135, 148–150]. Notable examples come from recent research on symptoms of depression and anxiety [149, 151]. Pentland et al. [149, 150] and Mohr et al. [148, 152] have demonstrated that passive sensing of GPS and other cell-phone usage metrics can be used to classify symptom severity. Beyond mere classification, Madan et al. [150] confirmed that Bluetooth-detected social interactions and movement patterns were associated, and that in some cases EMA-reported stress levels actually preceded stress-related behaviors such as travel to locations known to provoke anxiety.

Psychosocial stress is known to increase vulnerability to environmental pathogens [153, 154], but there has been less emphasis on the feedback loop whereby environmental exposures exacerbate stress and cognitive function more broadly. Air pollution exposure has been linked to symptoms of stress and depressed mood [155–159], raising the prospect that ecologic measurement of emotional well-being could be leveraged to investigate the cognitive impact of exposure to air pollution. For example, Bullinger et al. [158] implemented a geographically explicit daily diary protocol with small samples of healthy adults from polluted and non-polluted regions of Bavaria, assessing self-reported mood (including stress and “annoyance”) along with a range of ambient pollutant concentrations and weather conditions each day over a 2-month period. Though a small preliminary study, results suggested time-lagged effects of elevated pollutant exposure on depressed mood and elevated stress.

GEMA methods have also been utilized by physical activity researchers, although the focus of mobile survey reports in these studies have most often been restricted to behavioral assessments associated with physical activity patterns [84–86, 160–163]. Regardless, it is interesting to consider whether the same mechanisms believed to drive the well-known connection between physical activity and subjective well-being (e.g., cardio-pulmonary function) [164–168], may also explain the potentially detrimental impact of exposure to air pollution on mental health [154]. Studies are needed to explicitly measure the degree that a positive association between exercise and psychological well-being may be diluted by simultaneous exposure to particulate matter in the air. This is an example of a transdisciplinary research question involving multivariate geographic correlates that could be readily addressed with GEMA methods. The ability to systematically contrast geographic correlates that may produce countervailing effects with unknown population-level impact is among the key advances offered by the GEMA framework.

## Risk and resilience: neighborhood effects

Researchers and community stakeholders have long suspected that impoverished neighborhood conditions have a detrimental impact on the mental health of residents [169, 170]. Only recently have longitudinal studies begun to systematically document the way chronic exposure accrues within environments that are devoid of resources and periodically traumatic [110, 171, 172]. This literature operationalizes neighborhood disorder via systematic measurement of street-level conditions, including damage to buildings and other structures, litter, and criminal activity [173]. Viewed through this lens, retail outlets are not simply isolated drug-use cues or access points, but are elements of a broader ecologic system [146, 147, 152]. Alcohol retailers, for instance, cue and enable drinking, while they also potentiate deviant behaviors, interpersonal violence, and crime [169, 170, 174–177].

Longitudinal documentation of neighborhood disorder and mental health has revealed significant person-level variation in resilience to neighborhood stressors that is due to both the type and severity of stressors involved [178–180]. This familiar pattern is consistent with other ecological exposures work, once again highlighting the need for GEMA research designed to connect multi-level sources of information extending down from neighborhood conditions to ongoing subjective experience. It would be useful, for instance, to investigate the real-world circumstances under which residents experience feelings of safety, control, and freedom to improve their circumstances, all of which have been linked to protection against substance misuse and other mental health problems [181–185].

Ecological research on the effects of natural and man-made disasters on neighborhoods provides a contrasting perspective on neighborhood conditions and mental health, results of which suggest that the effects of severe residential damage are similar to effects of poverty and neighborhood disorder [186–188]. One study assessing the impact of Hurricane Sandy used tablets to conduct 1000 probability-based household surveys, randomly selected to represent the population of residents located within a geographic catchment extending along the entire coastal area affected by the storm. Findings indicate that children residing in homes that were damaged by the storm were particularly susceptible, with rates of depressive symptoms 2–4 times that of children from homes that incurred no damage [159]. It is noteworthy that researchers in this area often focus on psychological resilience and recovery [189, 190], and have developed instruments for assessing individual susceptibility to environmental adversity [22, 191], resources that may be useful to implement within GEMA research on the mental health of those in low-resource, disordered communities more broadly.

## Discussion

Advances in GEMA methods correspond with novel transdisciplinary “team” science approaches to mobile health (mHealth) research and practice more generally [192]. EMA, like any self-report method, is subject to the limitations of human respondents’ perceptual filters. People cannot report that which they are unaware [49], and what they do report is a subjective account of ecological context. Geographic momentary assessment (GMA) of real-time locations provides an objective relational connection to place, and thereby a rapidly expanding array of data, promising to transform environmental and health research and practice in the years to come.

Within a combined GEMA framework, relational data of this kind can enrich our perspective in absolute terms while also cross-validating EMA responses. EMA situational reports need not be relied on as objective ecologic measures, and instead can be valued precisely because they are free to diverge from other sources of cross-referenced ecologic data. National Oceanic and Atmospheric Association weather archives may indicate that it was “mostly sunny” on the afternoon of a certain day within a certain zipcode, but what is more interesting is the variation among subject reports at that same moment—some of whom, perhaps those who are characterologically pessimistic or just feeling down, may interpret the sky as “partly cloudy”.

## Methodological considerations

Careful consideration of the distinction between traditional EMA and GEMA reveals a number of implications that extend well beyond the basic idea that EMA surveys can be linked to geographic coordinates. First of all, because the inherent purpose of GEMA is research on the multivariate association between geographic correlates and subjective experience, researchers must consider sources of both real and spurious *spatial autocorrelation*, or the clustering of similar values as a function of their geographic proximity. Statistical inference in the presence of spatial autocorrelation requires valid information about the spatio-temporal distribution of the variables involved [193, 194]. Even when geographically explicit, a research protocol that only collects location information within EMA reports is blind by design to subjects’ baseline exposure to geographic correlates when EMA reports are *not* completed (i.e., the vast majority of the time). Without person-level data on the baserate of geographic exposures, inference regarding associated EMA reports can be misleading [195], for the same reasons that the predictive power of a diagnostic survey instrument (e.g., a brief depression screener) is directly related to the baseline prevalence of the disease within the group being tested. For example, given a hypothetical sample of depression ratings that are consistently elevated, drawn from two cities that have very different ambient air pollution levels, failure to account for spatial autocorrelation might lead one to conclude that air pollution is linked to depression in one city but not the other; that is, that air pollution and depression are associated in the polluted city, when in fact we know a priori that the subjects from both cities were equally depressed regardless.

One way EMA researchers have drawn inference about place-based, situational correlates is by contrasting random or otherwise time-based EMA reports (the within-subject control) with reports that are associated with a correlated event of interest (the within-subject case)—such as a drug-use event [140, 196, 197]. When an additional stream of data that is spatially distributed is incorporated it becomes necessary to evaluate the degree to which corresponding EMA reports are themselves spatially representative. Just as probability-based sampling ensures that opinion survey respondents are representative of the spatially distributed population from which they are drawn, ideally the timing and frequency of GEMA reports are reasonably proportionate to the underlying baserate that an individual spends time in the context of geographic correlates under study. GEMA protocols that include continuous geolocation monitoring provide complete geographic exposure information and thus the ability to account for person-level baserates. When necessary, GEMA methods can even be used to oversample EMA reports accordingly via real-time geographic survey-prompting procedures.

Data security and privacy will remain important considerations for the foreseeable future [198–201]. While the research subject consent process is central, the inherently identifiable nature of individual geographic mobility data presents new ethical challenges, particularly regarding participants’ access to, understanding of, and control over their own information [202]. Nonetheless, there are data security and processing methods that can substantially minimize risks by “masking” and ultimately removing geographic information altogether. Encryption and geographic masking procedures that remove geolocation precision from raw location data provide security protection during the data collection, processing, and analysis phases the research. Perhaps more importantly, location coordinates can be pre-processed in a way that allows selective extraction of research-relevant indicators while sensitive location information is stored separately and ultimately destroyed. For example, participants’ geographic location data can be used to generate exposure counts that are completely devoid of geographic or otherwise identifiable information. Thus, geographic precision can be tailored such that the spatial resolution of location coordinates is the minimum level required to address a research question deemed to have a societal impact that is significant and commensurate to the risks of the research.

## Conclusions

Not unlike the data on geographic variation in lifespan [1, 2], international surveillance data indicate that markers of well-being like subjective happiness are much more prevalent in some nations than others, varying significantly between municipal areas [203–205]. Geographically explicit, real-time reports of well-being are also being aggregated from social media such as Twitter, confirming patterns observed via conventional surveys [206–208]. More intriguing, geographically referenced photos shared on the Internet are emerging as a public health surveillance tool [209–211], and together, these new geographically-explicit indicators suggest that not only does subjective well-being vary as a function of place, but that the temporal dynamics of this association unfold differently over the course of the day [206, 207, 210].

For cultural anthropologists and geographers, as well as sociologists, urban planners, and others who specialize in the study of the way places have been developed by people, it is natural to focus on dynamic interactions between people and places, including the intersection of place and health. Yet the traditional frame of reference is spatially and temporally macro, documenting the relatively slow evolution of local health related-norms, for example, or the differential impact of large-scale public policies that unfold over years or decades. The existence of macro-level trends notwithstanding, the meso- and in some cases micro-level dynamic processes nested within the cyclic daily routines of individuals can increasingly be captured by modern technologies, and it is interesting to consider the degree to which variance in population health outcomes will be better accounted for by a geographically explicit measurement framework that weighs the reciprocal push and pull of both the macro and the micro [19, 212].

## Footnotes

1 This framework separates EMA-only and GMA-only research from GEMA studies that implement both. The combination of EMA and GPS tracking was referred to as GMA in one previous paper [17], which is included within the GEMA section of this review.
